# 5'UTR mutations of *ENG *cause hereditary hemorrhagic telangiectasia

**DOI:** 10.1186/1750-1172-6-85

**Published:** 2011-12-22

**Authors:** Kristy Damjanovich, Carmen Langa, Francisco J Blanco, Jamie McDonald, Luisa M Botella, Carmelo Bernabeu, Whitney Wooderchak-Donahue, David A Stevenson, Pinar Bayrak-Toydemir

**Affiliations:** 1 ARUP Institute for Clinical and Experimental Pathology, Salt Lake City, UT, USA; 2 Centro de Investigaciones Biológicas, CSIC and Centro de Investigacion Biomedica en Red de Enfermedades Raras (CIBERER), 28040 Madrid, Spain; 3 Department of Pathology, University of Utah, Salt Lake City, UT, USA; 4 Department of Pediatrics, University of Utah, Salt Lake City, UT, USA

**Keywords:** 5'UTR region, ENG, c.-127C > T, c.-9G > A, homozygous

## Abstract

**Background:**

Hereditary hemorrhagic telangiectasia (HHT) is a vascular disorder characterized by epistaxis, arteriovenous malformations, and telangiectases. The majority of the patients have a mutation in the coding region of the activin A receptor type II-like 1 (*ACVRL1*) or Endoglin (*ENG*) gene. However, in approximately 15% of cases, sequencing analysis and deletion/duplication testing fail to identify mutations in the coding regions of these genes. Knowing its vital role in transcription and translation control, we were prompted to investigate the 5'untranslated region (UTR) of *ENG*.

**Methods and Results:**

We sequenced the 5'UTR of *ENG *for 154 HHT patients without mutations in *ENG *or *ACVRL1 *coding regions. We found a mutation (c.-127C > T), which is predicted to affect translation initiation and alter the reading frame of endoglin. This mutation was found in a family with linkage to the *ENG*, as well as in three other patients, one of which had an affected sibling with the same mutation. *In vitro *expression studies showed that a construct with the c.-127C > T mutation alters the translation and decreases the level of the endoglin protein. In addition, a c.-9G > A mutation was found in three patients, one of whom was homozygous for this mutation. Expression studies showed decreased protein levels suggesting that the c.-9G > A is a hypomorphic mutation.

**Conclusions:**

Our results emphasize the need for the inclusion of the 5'UTR region of *ENG *in clinical testing for HHT.

## Background

Hereditary hemorrhagic telangiectasia (HHT) is an autosomal dominant vascular dysplasia characterized by epistaxis, telangiectasesand arteriovenous malformations (AVMs). AVMs that occur in the lungs, brain, or gastrointestinal tract can cause life-threatening complications secondary to either hemorrhage or the shunting of blood through abnormal blood vessels [1-5]. HHT is diagnosed on clinical grounds when an individual has three or more of the following diagnostic criteria: spontaneous- recurrent epistaxis, mucocutaneous telangiectases (especially on tongue, lips, oral mucosa, fingers, and nose), internal AVMs (pulmonary, cerebral, hepatic, gastrointestinal, spinal), and a first degree relative with HHT. The diagnosis is considered possible or suspected when two criteria are present and unlikely when there are fewer than two [6]. HHT is a clinically heterogeneous disorder, with symptoms often differing among family members, making the disorder difficult to diagnose [7,8].

HHT is a genetically heterogeneous disorder for which mutations in more than three genes cause disease. The majority of the clinically diagnosed HHT patients have a mutation in the coding regions of the Endoglin (*ENG*) gene or activin A receptor type II-like 1 (*ACVRL1*) gene [9-15], leading to HHT1 or HHT2, respectively. Mutations in *SMAD4 *have been detected in approximately 2% of patients with HHT and cause HHT and juvenile polyposis (JP/HHT) syndrome [16]. At least two additional genes, at 5q31.3-32 (HHT3) [17] and 7p14 (HHT4) [18], have been suggested by linkage studies.

Currently, molecular diagnosis of HHT involves sequencing of *ACVRL1 *and *ENG *coding regions, large deletion/duplication analysis, and if no mutation is identified, analysis of *SMAD4*. Approximately 15% of HHT cases have no mutations found in coding regions of these three genes [19,20]. But linkage studies in some of these families still implicate the *ENG *locus (PBT unpublished data). This is possible if mutations are in the noncoding regions such as introns or regulatory parts of the *ENG *gene. In particular, mutations in the 5'UTR may explain the pathogenesis of the disorder in some cases, since most of the transcription and/or translation protein complexes bind and regulate expression from the 5'UTR of the gene [21,22] Based on this information, combined with supportive linkage data to the *ENG*, we decided to investigate the role of the 5'UTR region of *ENG*. We sequenced this region in 154 unrelated HHT patients who do not carry a disease causing mutation in the coding region of the *ACVRL1 *and *ENG *genes by sequencing and deletion/duplication analyses.

## Methods

### Subjects

Our study group consists of 154 unrelated HHT cases. Cases included were those with two or more HHT clinical diagnostic criteria reported by their physician and negative mutation results. Information regarding HHT symptoms and manifestations was obtained from a disorder specific history form completed by ordering physicians and/or by assessment at the HHT Center at the University of Utah. Cases selected were negative for mutations by sequencing of the coding region and intron/exon boundaries, and also deletion/duplication analysis of the *ACVRL1 *and *ENG *genes. This study was approved by the Institutional Review Board of the University of Utah. The control group consisted of 134 healthy individuals. Based on the mutation results from the study group in Utah, a later collaboration was established with the Spanish HHT Genetics group to include one additional family with the c.-127C > T mutation. Although this family is not part of 154 patients' cohort, it has been included to provide additional clinical correlation for this mutation.

### Sequencing

Genomic DNA was extracted via automated Magna Pure (Roche Diagnostics, Indianapolis, IN) from whole blood. Primers were designed to amplify the entire 5'UTR region of *ENG*, BigDye sequencing chemistry was used to sequence the PCR products in both directions using the ABI 3730xl DNA analyzer (Applied Biosystems, Foster City, CA). The sequences were analyzed by the Mutation Surveyor program (Softgenetics, State College, PA). The variants detected were compared to the NCBI dbSNP databases to determine if the nucleotide change found in our study had previously been reported in healthy individuals.

### Plasmids

The 3-kb fragment of human endoglin cDNA cloned in pUC13 was previously described [23]. This plasmid was used as a template to generate the single endoglin mutants c.-9G > A and c.-127C > T. The mutant c.-9G > A was amplified using the following primers: 5'- AGGCCCCCACATGGACAGCAT -3'(Forward) and 5'-ATGCTGTCCATGTGGGGGCCT -3' (Reverse). The mutant c.-127C > T was amplified using the following primers: 5'- TGGAGCAGGGATGCCGTCGCT -3' (Forward) and 5'-AGCGACGGCATCCCTGCTCCA -3' (Reverse). The mutant c.-205A > C was amplified using the following primers: 5'- TTCGGACAGCCACTCCAGCCC - 3' (Forward) and 5'- GGGCTGGAGTGGCTGTCCGAA - 3' (Reverse). Then the single endoglin mutant c.-9G > A was used as a template to generate the double mutant c.-9G > A and c.1A > G in the presence of following primers: 5'- ATGGACAGCGTGGACCGCGGC -3' (Forward) and 5'-GCCGCGGTCCACGCTGTCCAT -3' (Reverse). The resulting pUC13 plasmids containing wild type and mutant sequences were digested with *EcoRI *and the 3-kb endoglin fragments were inserted into the pCEXV expression vector [23]. The expression vector 437/586-Endo encoding a hemagglutinin (HA)-tagged truncated version of human endoglin in pDisplay vector (Invitrogen) has been reported [24].

### Cell culture, transfections and western blot analyses

The monkey kidney COS-7 cell line was cultured in DMEM supplemented with 10% heat inactivated fetal calf serum, 2 mM L-glutamine, penicillin (100 U/ml), and gentamycin (25 mg/ml). For functional studies, cell transfections were carried out using SuperFect Reagent (Qiagen, Hilden, Germany) as vehicle for plasmids, according to the manufacturer's instructions. Cells were cotransfected with endoglin constructs in pCEXV and HA-437/586-Endo in pDisplay to correct for transfection efficiency. Twenty four hours after transfection, cells were lysed in lysis buffer and subjected to immunoblotting with anti-endoglin (clone P4A4; DSHB, University of Iowa), anti-HA (clone 12CA5; Boehringer Mannheim) or anti-actin (clone AC-15; Sigma) mouse monoclonal antibodies [24]. The presence of the specific proteins was revealed with horseradish peroxidase conjugated anti-mouse IgG (Dako, Barcelona, Spain) and the reaction was developed by addition of supersignal chemiluminescent substrate (Pierce, Thermo Scientific, Spain). Protein bands were visualized with a ChemiDoc™ XRS+ equipment (Bio-Rad, Madrid, Spain) and their intensity was quantified using Image Lab™ software.

## Results

To understand the role of 5'UTR of *ENG *we sequenced the noncoding region of exon 1 of *ENG *for 154 unrelated patients/probands with 2 or more clinical diagnostic criteria. These results revealed three sequence changes; c.-127C > T, c.-205A > C, and c.-9G > A. Out of 154, three had c.-127C > T, one had c.-205A > C, and three had c.-9G > A mutation. When available, probands' family members were studied to see if the mutation tracked with the disease. For the c.-127C > T mutation, we included a family from Northern Spain to provide additional information about this mutation. Clinical manifestations of the probands and their evaluated family members are summarized in Table 1. Cases listed as having no solid organ involvement had screening for pulmonary AVMs (PAVMs) by contrast echocardiogram and/or chest computed tomography (CT) and for brain AVMs by a contrasted magnetic resonance imaging (MRI), and physical examination and medical history that did not suggest other AVMs. Sequencing results of 134 healthy control samples did not reveal any sequence change in the 5'UTR of *ENG*.

**Table 1:** Molecular results and clinical findings in probands and family members

Family #	Individual (age at examination)	Molecular Result	Telangiectasia	Epistaxis	Solid Organ Involvement	Segregation in the family
Family 1	Proband (74 yo)	c.-127C > T	hands, lips, cheeks, palate, ears	6-8/day	GI telangiectases, PAVM	Yes
	Brother (74 yo)	c.-127C > T	hands, lips	2-3/day	GI telangiectases, PAVM	

Family 2	Proband (67 yo)	c.-127C > T	hands, ear, lips, tongue, palate, conjunctivae	1-2/month	none	Yes
	Daughter (50 yo)	c.-127C > T	lips, tongue, palate	1-2/week	PAVMs	
	Son (39 yo)	c.-127C > T	lips, palate, ears, hands	2/week	PAVM	
	Nephew (41 yo)	c.-127C > T	yes	yes	PAVM	
	Nephew (42 yo)	Negative for c.-127C > T	none	no	unknown	
	Grandniece (18 yo)	c.-127C > T	yes	daily	Spinal AVM	
	Grandnephew (8 yo)	c.-127C > T	unknown	daily	CAVM	

Family 3	Proband 3 (28 yo)	c.-127C > T	face	2-6/week	PAVM	No

Family 4	Proband 4 (41 yo)	c.-127C > T	multiple face, hands and feet	yes	PAVM	* NI
	Unaffected father of proband	Negative for c.-127C > T	none	none	unknown	
	Unaffected brother of proband	Negative for c.-127C > T	none	none	unknown	

Family 5	Proband 5 (10 yo)	c.-205A > C	hands and lip	1/month	none	Yes
	Brother (6 yo)	Negative for c.-205A > C	hands	1 every 2 months	none	
	Father (38 yo)	Negative for c.-205A > C	few on hand	1 every 2 months	unknown	
	Mother (38 yo)	c.-205A > C	none	none	unknown	

Family 6	Proband 6 (4 yo)	c.-9G > A	2 face, 1 hand	2/month (mild)	none	Yes
	Mother (26 yo)	c.-9G > A	4 hands, 1 face	2/month (mild)	none	
	Grandmother (52 yo)	c.-9G > A	lips, ear, face, hands	4/month (mild)	none	

Family 7	Proband 7 (27 yo)	c.-9G > A	few on face	only in childhood	unknown	No

Family 8	Proband 8 (78 yo)	c.-9G > A (homozygous)	lips, tongue, ear, hands, face and pharynx	daily	none	No
	Son (40 yo)	c.-9G > A (obligate carrier- but not tested)	lips, face	"regular", "bad"	none	

PAVM: Pulmonary AVM

CAVM: Cerebral AVM

GI: Gastrointestinal

Unknown: has not been tested

*NI: Not informative. Based on family history it is possible that this mutation segregates in the family. However, a* de novo *event cannot be excluded.

**Figure 1:**
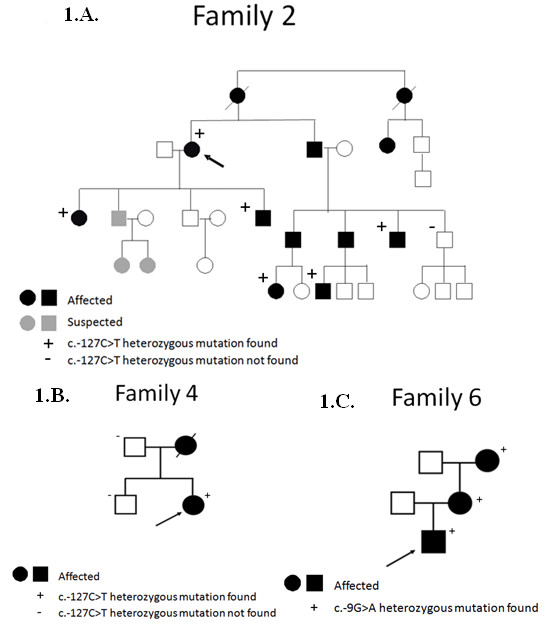
**A. Family segregation study for family 2**. The pedigree for family 2 is shown. The c.-127C > T mutation was shown to segregate among affected individuals in this family, where 5 clinically affected family members were available for the family segregation study. **1B. Family segregation study for family 4**. The pedigree for family 4 is shown. Three family members were sequenced. Two unaffected family members were shown to be negative for the mutation. **1C. Family segregation study for family 6**. The pedigree for family 6 is shown. 3 family members were available from family 6. All 3 carried the -127C > T mutation.

The c.-127C > T heterozygous change was found in three out of the 154 clincal HHT cases and in one case from Northern Spain (family 4). For one of these cases (family 1), an affected sibling was also available and was found to be positive for the mutation. Two of the siblings met clinical diagnostic criteria with frequent epistaxis, typical telagiectasia, PAVMs, and gastrointestinal (GI) telangiectasia. The second patient (proband 2) was a member of a family (family 2) linked to the *ENG *by locus specific short tandem repeat (STR) markers. The *ACVRL1 *region was excluded in this family (data not shown). There were 5 clinically affected family members available for the family segregation study (Figure 1a). The mutation is carried by all studied family members affected with HHT. Although our 67 year old proband did not have any solid organ involvement, an 18 year old grandniece had a spinal AVM, an 8 year old grandnephew had cerebral AVMs (CAVMs), and three other affected family members had pulmonary AVMs (PAVMs). Family members of the third patient (proband 3) were not available for family segregation study. Proband 3 had PAVMs, telangiectases on the face and 2-6 episodes/week of epistaxis. The c.-127C > T mutation was also found in one family proband from Northern Spain (family 4). No other affected family member, including his deceased mother, was available for a segregation study. But the mutation was not seen in his unaffected father or brother (Figure 1b).

**Figure 2:**
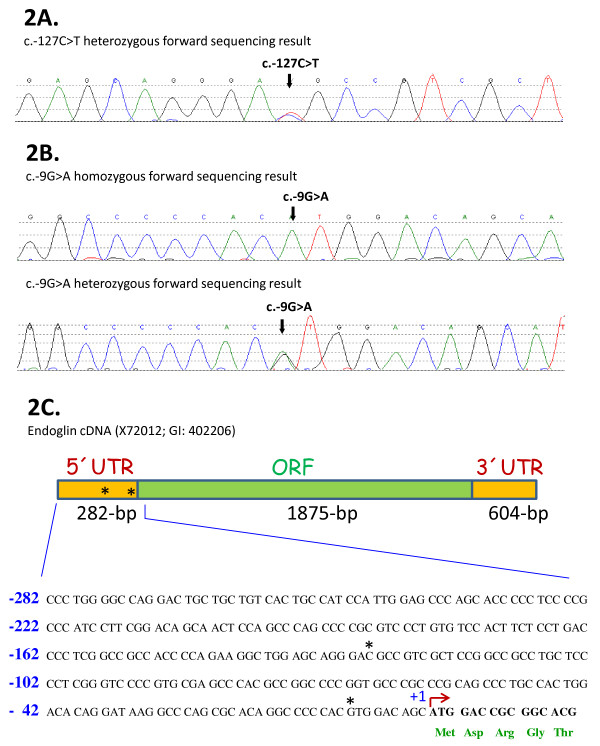
**A. Sequencing results from one individual with the c.-127C > T heterozygous mutation**. The forward sequence is shown. The arrow indicates the position of the mutation. **2B. Sequencing results from two individuals (one homozygous and one heterozygous) for the -9G > A mutation**. The arrow indicates the position of the mutation. **2C. Schematic representation of endoglin mRNA. The 5'UTR, the 3'UTR and the open reading frame (ORF) are indicated**. Endoglin cDNA accession number (X72012) corresponding to the 3073-bp mRNA of endoglin [21] and the gene ID (GI, 402206) are also included. The sequence of the 5'UTR and part of the signal peptide (-282/+15) is shown. Asterisks indicate the positions mutated in panels 2a and 2b.

**Figure 3:**
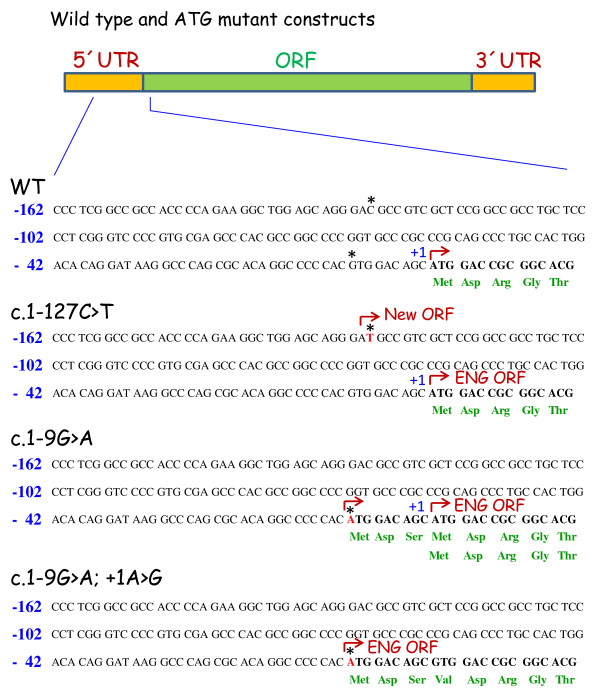
**Schematic representation of wild type and mutant versions of endoglin**. The 5'UTR, the 3'UTR and the region corresponding to the ORF are indicated (top). The sequence of wild type (WT) and mutants (c.-127C > T; c.-9G > A; c.-9G > A and +1A > G) corresponding only to the -162/+15 region is shown. Asterisks indicate the positions of the HHT mutations of Figs. 2a and 2b. The constitutive translation initiation (+1), the predicted translated amino acids (three letters code in green) as well as the putative translation initiation sites (brown broken arrow) are also indicated.

**Figure 4:**
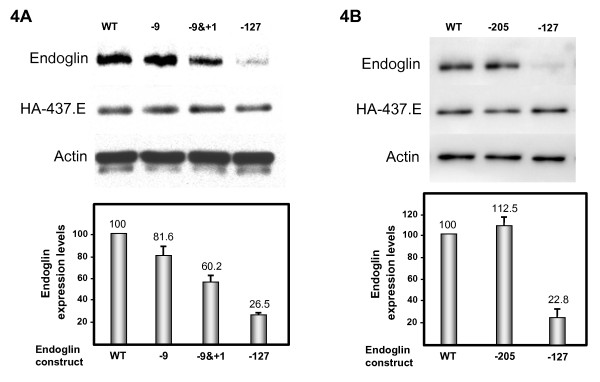
**Functional analysis of ATG endoglin mutants**. COS-7 cells were transfected with wild type (WT) or endoglin mutants 4A: c.-9G > A (-9), c.-9G > A & c.+1A > G (-9&+1) or c.-127C > T (-127), and 4B: c.-205A > C (-205) in the presence of HA-437/586-Endo in pDisplay (HA-437.E) to correct for transfection efficiency. After 24 h, cells were lysed and total cell lysates were subjected to western blot analysis using anti-endoglin, anti-HA or anti-actin antibodies. As a loading control, the presence of actin is included. Normalized endoglin levels relative to cotransfected HA-437/586-Endo and total actin proteins are shown in the histogram. An arbitrary value of 100 was assigned to WT endoglin. The average of six different experiments is shown in each panel.

The sequence ideogram and neighboring sequences of the c.-127C > T mutation are shown in Figure 2a. This sequence alteration creates a potential AUG initiation codon at base -127 from translation initiation of the *ENG *gene. The c.-127C > T change is not reported in the NCBI dbSNP database. NetStart 1.0 Prediction Program [25] predicts that this mutation creates a new translation start site (TIS) with an altered reading frame (Figures 2c and 3). Interestingly, the sequence surrounding the new TIS fits well with the Kozak consensus and other motifs that play a major role in the initiation of the translation process [26,27], suggesting that this new TIS may be functionally active. Because translation usually initiates solely at the first ATG codon in an adequate context, it is likely that the new TIS at -126/-128 is competing advantageously with the constitutive TIS at +1. To test this hypothesis, we generated a mutant construct in a full length endoglin cDNA, that contains the 5'UTR [23], where the c.-127C > T change was introduced (Figure 3). The wild type and mutant constructs were cloned into an expression vector and the levels of endoglin protein expression were assessed by transient transfection in the monkey cell line COS-7. As shown in Figure 4, we found that protein expression levels of the mutant endoglin construct c.-127C > T were markedly reduced (74%) with respect to the wild type construct. This result suggests that the c.-127C > T mutation generates a functional TIS out of frame that interferes with translation initiation of the constitutive ATG at +1, leading to endoglin haploinsufficiency.

The c.-9G > A mutation (Figure 2b) was found in three HHT families. Three family members were available from family 6 (Figure 1c). All carried the mutation and had infrequent epistaxis and telangiectases with no solid organ involvement. Proband 7 was 27 years old when she was examined. She had a few telangiectases on her face and infrequent epistaxis only during her childhood. She did not have any solid organ involvement. There were no other family members available for this study. All of these patient's clinical findings were relatively mild, none of them had solid organ involvement or severe/frequent epistaxis causing anemia or blood transfusion.

Proband 8 was found to be homozygous for c.-9G > A mutation (Figure 2b). In order to confirm this result and to rule out primer binding site polymorphisms, this region was sequenced with three different primer sets. Multiplex ligation dependent probe amplification (MLPA) method was used to test for a large deletion of the region. MLPA results and sequencing with different primer sets confirmed that proband 8 carries two copies of the mutant allele for this region (data not shown).

Proband 8 had daily epistaxis and telangiectases on his lips, tongue, ear, hands, face and pharynx. His son's clinical findings were not as profound as his father. He had a few telangiectases on his lips and face, and epistaxis. Neither of them had solid organ involvement. His son was not available for molecular testing; however, he is an obligate carrier for the mutation as his father is homozygous. The parents of the proband were deceased, thus not available for examination or testing. Neither was known by the proband to have nosebleeds or telangiectasia. The mother reportedly died at age 42 of tuberculosis and father of a myocardial infarct in the decade of his 60s.

The c.-9G > A mutation is not reported in NCBI dbSNP database, nor it was seen in our control group. This mutation is predicted to create a new TIS with the same reading frame as endoglin and a resulting protein which contains three additional amino acids in the leader sequence (Figure 3). Of note, the length of the resulting leader sequence (28 amino acids) is within the normal size range of cleavable amino terminal signal peptides in precursor proteins [28]. To assess whether the new TIS at -9 was affecting the expression of the endoglin protein, we generated two different mutant constructs in a full length endoglin cDNA that contains the 5'UTR. A single mutant contained the c.-9G > A change of the HHT patients, while a double mutant contained both the c.-9G > A mutation plus the c.1A > G mutation. Functional studies of the c.-9G > A mutation showed that expression of the mutant protein was reduced approximately 20% compared to the wild type protein (Figure 4A). To prove that the ATG at -9 was functioning as a real TIS, the constitutive initiation site at position 1 was abolished in the double mutant (c.-9G > A and c.1A > G). Thus, transfection studies with this double mutant demonstrated that the corresponding endoglin protein was expressed, although at a much lower level (60%), as compared to 81% with the c.-9G > A construct. While the predicted mature endoglin protein driven by the TIS at -9 is identical to the one driven by the constitutive TIS at +1, the decreased expression levels of endoglin with the c.-9G > A mutation are likely due to a lower translation efficiency and/or a less efficient processing of the endoglin precursor protein through the secretory pathway. Taken together, these results suggest that the mutation c.-9G > A confers slightly reduced expression of the mutant protein that is compatible with the mild effect in heterozygosis and a more severe, but still classical, HHT phenotype in homozygosis.

The c.-205A > C heterozygous variant was found in one patient (Proband 5). Three family members were available for study. It was not found in a brother and father with infrequent, recurring nosebleeds and telangiectases in characteristic locations, but was identified in an asymptomatic mother. This variant was not found in dbSNP database, nor was it found in healthy controls. However, based on *in silico *analyses this variant is not predicted to have a significant effect on the regulation of the translation or transcription. In the family segregation study this mutation does not track with symptoms of HHT, and *in silico *analysis does not support pathogenicity. Expression analysis of the mutant construct (c.-205A > C) showed similar protein levels as the wild type construct (Figure 4B) confirming that it is a benign sequence change.

## Discussion

HHT is a genetically heterogeneous disease with at least three causative genes [9-15]. 15% of clinically diagnosed HHT cases cannot be explained by mutations in the coding regions or exon/intron junctions of *ACVRL1*, *ENG*, or *SMAD4 *[19,20]). Yet in some families, linkage data suggests *ACVRL1 *or *ENG *to be the causative gene. Therefore, non-coding regions may play a role in the disease. However, previously described mutations in *ENG *were located only on the coding regions and exon-intron junctions of the gene [29,30]. So far, no 5'UTR mutations or deep intronic mutations have been described. *ENG *promoter activity was found to be within the upstream 400 bp region from the TIS, and an area near the transcription initiation site of *ENG *was determined to be essential for promoter function [21,31]. We therefore chose this critical region to analyze in our unexplained HHT cases. We have identified a 5'UTR mutation (c.-127C > T) in 3 unrelated probands, 2 of which had family members evaluated for co-segregation studies. One family with the same mutation from Northern Spain is also included in this study for additional clinical description of the mutation. All affected individuals had classical clinical findings of HHT disease, including many with solid organ involvement. The clinical findings and medical histories in these four families are typical of HHT1 families previously reported [32]. The number of PAVMs observed in these families with c.-127C > T mutation seems possibly greater than typical for HHT1 disease, this may represent ascertainment bias since the majority of these patients were seen in an HHT specialty clinic that tend to attract patients with pulmonary AVMs for treatment and expected variation of HHT. During the revision of this manuscript, Kim et al reported a Korean family with the c.-127C > T mutation, in which the proband of the family has epistaxis and PAVM [33].

Current data suggest that most disease-causing mutations in *ENG *result in haploinsufficiency [8,32,34-36]. Thus, HHT is assumed to result from lack of sufficient protein for normal function [37]. Mutations resulting in structural alterations by misfolding and intracellular degradation of these proteins lead to lack of surface expression of the mutant proteins. The c.-127C > T mutation in the 5'UTR creates a new TIS resulting in an out-of-frame product. Translation initiation from this novel start site predicts prematurely truncated protein with no homology to wild type protein. This mutation effect would be similar to any frameshift mutations seen in the *ENG*, which is lack of the protein expression on the cell surface. Expression studies confirmed that endoglin protein level is decreased to 26% of the wild-type construct, a figure compatible with quantitative measurements of endoglin levels in endothelial cells derived from HHT1 patients [37]. Kozak sequences are conserved sequences that ribosomes recognize as the start of translation of the protein [26,27]. The original TIS of the *ENG *gene does not have a strong Kozak consensus sequence. This and the fact that translation preferentially initiates at the first ATG codon suggest that the new TIS is competing advantageously with the constitutive TIS at +1. 5' UTR mutations that change the initiation codon have been reported as disease causing mutations for other disorders [38,39]. However, our study provides the first functional evidence that 5'UTR of the *ENG *cause HHT.

The second sequence change found in this study is c.-205A > C. This variant does not affect the ATG translation initiation. There is no specific sequence in the endoglin promoter affected by this mutation based on *in silico *studies. Family segregation study also suggests that c.-205A > C is a benign sequence change (Table 1). Moreover, studies with the -205A > C mutant construct confirmed that this variant does not affect the expression level of ENG protein.

The c.-9G > A mutation has been found in three probands, one of whom is homozygous for the mutation. Proband 6, her mother and proband 7 were heterozygous for this mutation with mild clinical findings and no organ involvement. Proband 8 with a homozygous mutation had symptoms of HHT typical for a 78 year old heterozygous mutation carrier. It might be speculated that heterozygotes with this mutation might be more mildly affected than typical HHT patients. Although none of the heterozygous probands or affected family members was found to have solid organ involvement, no conclusion can be made from this small number of cases as to whether the epistaxis or oral/dermal telangiectases resulting from this mutation are more mild than typical.

The c.-9G > A mutation also creates a new TIS, yet does not alter the reading frame. Based on viability in the homozygous state, we suggest that the c.-9G > A mutation results in reduced, but not absent, protein production or function. The double construct study supports that the c.-9G > A mutation does not create a strong TIS and the existing TIS is also being used for translation. This confirms the leakiness of the initiation of the translation. Given the possibly milder phenotype in heterozygous patients, and viability in a homozygote patient, we conclude that the c.-9G > A mutation may represent a milder HHT mutation, which has never been reported before.

In addition to the consequences in translation, the pathogenic mutations at c.-9 and c.-127 may also have effects in the transcriptional regulation of Endoglin. In this sense, an *in silico *analysis using the MatInspector program revealed that several putative consensus motifs for transcription factors were either destroyed or generated (see Additional File 1). More specifically, the mutation c.-127C > T generates the disappearance of consensus motifs for WHNF and EGRF family members, while raises a new motif for the general transcription factor IID. Moreover, the mutation c.-9G > A generates the disappearance of several binding sites for EGRF, HESF, EBOX, HIF or p53 family members, while raises several motifs for p53, GCMF or SRFF transcription factors. Finally, it is worth mentioning that many mutations leading to frameshift and truncation may result in nonsense mediated decay and therefore reduced mRNA levels [40]. In addition to the effects on protein translation/processing analyzed here, we cannot rule out the possibility that these ATG mutations may also decrease mRNA stability, as previously described in HHT1 for several truncation mutations of *ENG *[41].

## Conclusions

This study highlights two novel mutations in the 5'UTR region of the *ENG *gene, c.-9G > A and c.-127C > T. *In vitro *expression studies predict that these mutations would result in reduced expression of the endoglin protein. Taken together, the clinical, co-segregation and functional data suggest these mutations cause HHT in the families studied.

A 78 year old with HHT who is shown to be homozygous for c.-9G > A suggests that this mutations causes a leakiness of transcription initiation and possibly a milder clinical phenotype. This is the first report of an apparently pathogenic mutation found in the homozygous state in a patient with HHT. In summary, we detected mutations in the 5'UTR region of the *ENG *gene in 8 of 154 unrelated patients with known or suspected HHT in whom sequencing of the coding region and intron/exon border region of *ENG *and *ACVRL1 *had failed to identify a mutation. Analysis of the two mutations at the protein level found in seven probands suggests the involvement of the mutations in the pathogenesis of HHT. The 5'UTR of the *ENG *gene should be included in genetic testing for HHT to increase clinical sensitivity.

## Competing interests

The authors declare that they have no competing interests.

## Authors' contributions

KD and LMB carried out the molecular genetic studies. CL and FB carried out the functional studies. WWD performed the linkage analysis studies. DS and JM provided clinical evaluation/information. KD, PBT, CB, and JM wrote the manuscript. All authors read and approved the final manuscript.

## Supplementary Material

Additional file 1 **Search results for the mutations of the endoglin promoter in MatInspector**. The pathogenic mutations at c.-9G > A and c.-127C > T may also have effects in the transcriptional regulation of endoglin. *In silico *analysis using the MatInspector program revealed that several putative consensus motifs for transcription factors were either destroyed or generated. Consensus prediction is indicated by a 'Y' or 'N.' Click here for file

## References

[B1] Bayrak-Toydemir P Mao R Lewin S McDonald J Hereditary hemorrhagic telangiectasia: an overview of diagnosis and management in the molecular era for clinicians Genet Med 2004 48 12 175 91 10.1097/01.gim.0000132689.25644.7c 15266205

[B2] Abdalla SA Cymerman U Rushlow D Chen N Stoeber GP Lemire EG Letarte M Novel mutations and polymorphisms in genes causing hereditary haemorrhagic telangeictasia Hum Mutat 2005 25 320 321 15712271 10.1002/humu.9312

[B3] Giovani FS Sholvin CL Hereditary haemorrhagic telangiectasia: a clinical and scientific review Eur J Hum Genet 2009 17 860 871 10.1038/ejhg.2009.35 19337313 PMC2986493

[B4] Berg J Porteous M Reinhardt D Gallione C Holloway S Umasunthar T Lux A McKinnon W Marchuk D Guttmacher A Hereditary haemorrhagic telangiectasia: a questionnaire based study to delineate the different phenotypes casued by endoglin and ALK1 mutations J Med Genet 2003 40 585 590 10.1136/jmg.40.8.585 12920067 PMC1735540

[B5] Guttmacher AE Marchuk DA White RI Jr Hereditary hemorrhagic telangiectasia N Engl J Med 1995 333 918 924 10.1056/NEJM199510053331407 7666879

[B6] Shovlin CL Guttmacher AE Buscarini E Faughnan ME Hyland RH Westerman CJJ Kjeldsen AD Plauchu H Diagnostic criteria for hereditary hemorrhagic telangiectasia (Rendu-Osler-Weber syndrome) Am J Med Genet 2000 91 1 66 67 10.1002/(SICI)1096-8628(20000306)91:1<66::AID-AJMG12>3.0.CO;2-P 10751092

[B7] Faughnan ME Palda VA Garcia-Tsao G Geisthoff UW McDonald J Proctor DD Spears J Brown DH Buscarini E Chesnutt MS Cottin V Ganguly A Gossage JR Guttmacher AE Hyland RH Kennedy SJ Korzenik J Mager JJ Ozanne AP Piccirillo JF Picus D Plauchu H Porteous ME Pyeritz RE Ross DA Sabba C Swanson K Terry P Wallace MC Westermann CJ White RI Young LH Zarrabeitia R HHT Foundation International - Guidelines Working Group. International guidelines for the diagnosis and management of hereditary haemorrhagic telangiectasia J Med Genet 2011 48 2 73 87 10.1136/jmg.2009.069013 19553198

[B8] Shovlin CL Hereditary haemorrhagic telangiectasia: pathophysiology, diagnosis and treatment Blood Rev 2010 24 6 203 19 10.1016/j.blre.2010.07.001 20870325

[B9] Bossler AD Richards J George C Godmillow L Ganguly A Novel mutations in ENG and ACVRL1 identified in a series of 200 individuals undergoing clinical genetic testing for hereditary hemorrhagic telangiectasia (HHT): correlation of genotype with phenotype Hum Mutat 2006 57 7 667 75 10.1002/humu.20342 16752392

[B10] Fernandez-L A Sanz-Rodriguez F Zarrabeitia R Perez-Molino A Morales C Restrepo CM Ramirez JR Coto E Lenato GM Bernabeu C Botella LM Mutation study of Spanish patients with hereditary hemorrhagic telangiectasia and expression analysis of Endoglin and ALK1 Hum Mutat 2006 27 3 295 16470589 10.1002/humu.9413

[B11] Fontalba A Fernandez-L A García-Alegria E Albiñana V Garrido-Martin EM Blanco FJ Zarrabeitia R Perez-Molino A Bernabeu-Herrero ME Ojeda ML Fernandez-Luna JL Bernabeu C Botella LM Mutation study of Spanish patients with hereditary hemorrhagic telangiectasia BMC Med Genet 2008 9 75 18673552 10.1186/1471-2350-9-75 PMC2518546

[B12] McDonald J Damjanovich K Millson A Wooderchak W Chibuk JM Stevenson DA Gedge F Bayrak-Toydemir P Molecular diagnosis in hereditary hemorrhagic telangiectasia: findings in a series tested simultaneously by sequencing and deletion/duplication analysis Clin Genet 2011 79 4 335 44 10.1111/j.1399-0004.2010.01596.x 21158752

[B13] Lesca G Genin E Blachier C Olivieri C Coulet F Brunet G Dupuis-Girod S Buscarini E Soubrier F Calender A Danesino C Giraud S Plauchu H French-Italian HHT Network Hereditary hemorrhagic telangiectasia: evidence for regional founder effects of ACVRL1 mutations in French and Italian patients Eur J Hum Genet 2008 16 6 742 9 10.1038/ejhg.2008.3 18285823

[B14] McAllister KA Grogg KM Johnson DW Gallione CJ Baldwin MA Jackson CE Helmbold EA Markel DS McKinnon WC Murrell J McCormick MK Pericak-Vance MA Heutink P Oostra BA Haitjema T Westerman CJJ Porteous ME Guttmacher AE Letarte M Marchuk DA Endoglin, a TGF-beta binding protein of endothelial cells, is the gene for hereditary haemorrhagic telangiectasia type 1 Nat Genet 1994 8 345 51 10.1038/ng1294-345 7894484

[B15] Johnson DW Berg JN Baldwin MA Gallione CJ Marondel I Yoon SJ Stenzel TT Speer M Pericak-Vance MA Diamond A Buttmacher AE Jackson CE Attisano L Kucherlapati R Porteous ME Marchuk DA Mutations in the activin receptor-like kinase 1 gene in hereditary hemorrhagic telangiectasia type 2 Nat Genet 1996 13 2 189 95 10.1038/ng0696-189 8640225

[B16] Gallione CJ Repetto GM Leguis E Rustgi AK Schelley SL Tejpar S Mitchell G Drouin E Westermann CJ Marchuk DA A combined syndrome of juvenile polyposis and hereditary hemorrhagic telangiectasia associated with mutations in MADH4 (SMAD4) Lancet 2004 363 9412 852 9 10.1016/S0140-6736(04)15732-2 15031030

[B17] Cole SG Begbie ME Wallace GMF Shovlin CL A new locus for hereditary hemorrhagic telangiectasia (HHT3) maps to chromosome 5 J Med Genet 2005 42 577 582 10.1136/jmg.2004.028712 15994879 PMC1736109

[B18] Bayrak-Toydemir P McDonald J Akarsu N Toydemir RM Calderon F Tuncali T Tang W Miller F Mao R A fourth locus for hereditary hemorrhagic telangiectasia maps to chromosome 7 Am J Med Genet Part A 2006 140A 20 2155 62 10.1002/ajmg.a.31450 16969873

[B19] Prigoda-Lee NL Savas S Abdalla SA Piovesan B Rushlow D Vandezande K Zhang E Ozcelik H Gallie BL Letarte M Hereditary haemorrhagic telangiectasia: mutation detection, test sensitivity and novel mutations J Med Genet 2006 43 9 722 8 10.1136/jmg.2006.042606 16690726 PMC2564570

[B20] McDonald J Bayrak-Toydemir P Pyeritz RE Hereditary hemorrhagic Telangiectasia: An overview of diagnosis, management, and pathogenesis Gen in Med 2011 13 7 607 16 10.1097/GIM.0b013e3182136d32 21546842

[B21] Rius C Smith JD Almendro N Langa C Botella LM Marchuk DA Vary CPH Bernabeu C Cloning of the promoter region of human endoglin, the target gene for hereditary hemorrhagic telangiectasia type 1 Blood 1998 92 12 4677 4690 9845534

[B22] Wilkie GS Dickson KS Gray NK Regulation of mRNA translation by 5'- and 3'-UTR-binding factors Trends Biochem Sci 2003 28 4 182 188 10.1016/S0968-0004(03)00051-3 12713901

[B23] Bellon T Corbi A Lastres P Cales C Cebrian M Vera S Chiefetz S Massague J Letarte M Bernabeu C Identification and expression of two forms of the human transforming growth factor-beta-binding protein endoglin with distinct cytoplasmic regions Eur J Immunol 1993 23 2340 2345 10.1002/eji.1830230943 8370410

[B24] Guerrero-Esteo M Sanchez-Elsner T Letamendia A Bernabeu C Extracellular and cytoplasmic domains of endoglin interact with the transforming growth factor-beta receptors I and II J Biol Chem 2002 277 32 29197 209 10.1074/jbc.M111991200 12015308

[B25] Pedersen AG Nielsen H Neural network prediction of translation initiation sites in eukaryotes: perspectives for EST and genome analysis ISMB 1997 226 233 9322041

[B26] Kozak M Regulation of translation via mRNA structure in prokaryotes and eukaryotes Gene 2005 361 13 37 16213112 10.1016/j.gene.2005.06.037

[B27] Kozak M At least six nucleotides preceding the AUG initiator codon enhance translation in mammalian cells J Mol Biol 1998 196 947 950 10.1016/0022-2836(87)90418-9 3681984

[B28] Zimmermann R Eyrisch S Ahmad M Helms V Protein translocation across the ER membrane Biochim Biophys Acta 2011 1808 3 912 24 10.1016/j.bbamem.2010.06.015 20599535

[B29] Richards-Yutz R Grant K Chao EC Walther SE Ganguly A Update on molecular diagnosis of hereditary hemorrhagic telangiectasia Hum Genet 2010 128 1 61 77 10.1007/s00439-010-0825-4 20414677

[B30] Lesca G Burnichon N Raux G Tosi M Pinson S Marion MJ Babin E Gilbert-Dussardier B Riviere S Giozet C Faivre L Plauchu H Frebourg T Calender A French Rendu-Osler Network Distribution of ENG and ACVRL1 (ALK1) mutations in French HHT patients Hum Mutat 2006 27 6 598 612 16705692 10.1002/humu.9421

[B31] Graulich W Nellelbeck DM Fischer D Kissel T Muller R Cell type specificity of the human endoglin promoter Gene 1999 227 1 55 62 10.1016/S0378-1119(98)00585-X 9931433

[B32] Cymerman U Vera S Karabegovic A Adballa S Letarte M Characterization of 17 novel endoglin mutations associated with hereditary hemorrhagic telangiectasia Hum Mutat 2003 21 482 492 10.1002/humu.10203 12673790

[B33] Kim MJ Kim ST Lee HD Lee KY Seo J Lee JB Lee YJ Oh SP Clinical and genetic analysis of three Korean families with hereditary hemorrhagic telangiectasia BMC Med Genet 2011 12 130 10.1186/1471-2350-12-130 21967607 PMC3202234

[B34] Pece-Barbara N Cymerman U Vera S Marchuk DA Letarte M Expression analysis of four endoglin missense mutations suggests that haploinsufficiency is the predominant mechanism for hereditary hemorrhagic telangiectasia type I Hum Mol Gen 1999 8 12 2171 2181 24 10.1093/hmg/8.12.2171 10545596

[B35] Gallione CJ Klaus DJ Yeh EY Stenzel TT Xue Y Anthony KB McAllister KA Baldwin MA Berg JN Lux A Smith JD Vary CPH Craigen WJ Westermann CJJ Warner ML Miller YE Jackson CE Guttmacher AE Marchuk DA Mutation expression analysis of the endoglin gene in hereditary hemorrhagic telangiectasia reveals null alleles Hum Mutat 1998 11 286 294 10.1002/(SICI)1098-1004(1998)11:4<286::AID-HUMU6>3.0.CO;2-B 9554745

[B36] Pece N Vera S Cymerman U White RI Wrana JL Letarte M Mutant endoglin in hereditary hemorrhagic telangiectasia type 1 is transiently expressed intracellularly and is not a dominant negative J Clin Invest 1997 100 10 2568 2579 10.1172/JCI119800 9366572 PMC508458

[B37] Abdalla SA Letarte M Hereditary haemorrhagic telangiectasia: current views on genetics and mechanisms of disease J Med Genet 2006 43 2 97 110 15879500 10.1136/jmg.2005.030833 PMC2603035

[B38] Matthes T Aguilar-Martinez P Pizzi-Bosman L Darbellay R Rubbia-Brandt L Giostra E Michel M Ganz T Beris P Severe hemochromatosis in a Portuguese family associated with a new mutation in the 5'-UTR of the *HAMP *gene Blood 2004 104 2181 2183 10.1182/blood-2004-01-0332 15198949

[B39] Lui L Dilworth D Gao L Monzon J Summers A Lassam N Hogg D Mutation of the *CDKN2A *5' UTR creates an aberrant initiation codon and predisposes to melanoma Nat Genet 1999 21 1 128 132 10.1038/5082 9916806

[B40] Khajavi M Inoue K Lupski JR Nonsense-mediated mRNA decay modulates clinical outcome of genetic disease Eur J Hum Genet 2006 14 10 1074 81 10.1038/sj.ejhg.5201649 16757948

[B41] Shovlin CL Hughes JM Scott J Seidman CE Seidman JG Characterization of endoglin and identification of novel mutations in hereditary hemorrhagic telangiectasia Am J Hum Genet 1997 61 1 68 79 10.1086/513906 9245986 PMC1715873

