# Supplementary figures and images for: 5'UTR mutations of ENG cause hereditary hemorrhagic telangiectasia
Source: Orphanet J Rare Dis. 2011 Dec 22;6:85. doi: 10.1186/1750-1172-6-85 (PMC3277489; doi:10.1186/1750-1172-6-85)

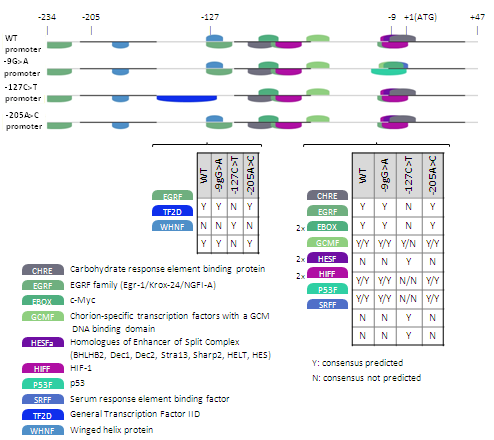

Supplement: Additional file 1 — Search results for the mutations of the endoglin promoter in MatInspector. The pathogenic mutations at c.-9G > A and c.-127C > T may also have effects in the transcriptional regulation of endoglin. In silico analysis using the MatInspector program revealed that several putative consensus motifs for transcription factors were either destroyed or generated. Consensus prediction is indicated by a 'Y' or 'N.' [file 1750-1172-6-85-S1.TIFF]
